# Myocardial deformation in patients with a single left ventricle using 2D cardiovascular magnetic resonance feature tracking: a case–control study

**DOI:** 10.1007/s10554-021-02230-2

**Published:** 2021-03-31

**Authors:** Fabian Strodka, Jana Logoteta, Roman Schuwerk, Mona Salehi Ravesh, Dominik Daniel Gabbert, Anselm Sebastian Uebing, Sylvia Krupickova, Inga Voges

**Affiliations:** 1 Department of Congenital Heart Disease and Paediatric Cardiology, University Hospital Schleswig-Holstein, Campus Kiel, Arnold-Heller-Str. 3, 24105 Kiel, Germany; 2 Department of Paediatric Cardiology, Children’s Hospital of Zurich, Zurich, Switzerland; 3 Department of Radiology and Neuroradiology, University Hospital Schleswig-Holstein, Kiel, Germany; 4 Royal Brompton Hospital, London, UK

**Keywords:** Single ventricle, Cardiovascular magnetic resonance, Feature tracking, Speckle tracking echocardiography

## Abstract

Ventricular dysfunction is a well-known complication in single ventricle patients in Fontan circulation. As studies exclusively examining patients with a single left ventricle (SLV) are sparse, we assessed left ventricular (LV) function in SLV patients by using 2D-cardiovascular magnetic resonance (CMR) feature tracking (2D-CMR-FT) and 2D-speckle tracking echocardiography (2D-STE). 54 SLV patients (11.4, 3.1–38.1 years) and 35 age-matched controls (12.3, 6.3–25.8 years) were included. LV global longitudinal, circumferential and radial strain (GLS, GCS, GRS) and strain rate (GLSR, GCSR, GRSR) were measured using 2D-CMR-FT. LV volumes, ejection fraction (LVEF) and mass were determined from short axis images. 2D-STE was applied in patients to measure peak systolic GLS and GLSR. In a subgroup analysis, we compared double inlet left ventricle (DILV) with tricuspid atresia (TA) patients. The population consisted of 19 DILV patients, 24 TA patients and 11 patients with diverse diagnoses. 52 patients were in NYHA class I and 2 patients were in class II. Most SLV patients had a normal systolic function but median LVEF in patients was lower compared to controls (55.6% vs. 61.2%, p = 0.0001). 2D-CMR-FT demonstrated reduced GLS, GCS and GCSR values in patients compared to controls. LVEF correlated with GS values in patients (p < 0.05). There was no significant difference between GLS values from 2D-CMR-FT and 2D-STE in the patient group. LVEF, LV volumes, GS and GSR (from 2D-CMR-FT) were not significantly different between DILV and TA patients. Although most SLV patients had a preserved EF derived by CMR, our results suggest that, LV deformation and function may behave differently in SLV patients compared to healthy subjects.

## Introduction

Since the introduction of the Fontan operation, life expectancy of single ventricle (SV) patients steadily improved, and an increasing number of SV patients is reaching adolescence and adulthood [1–3]. Despite this success, we must remind ourselves that the Fontan procedure is a palliative approach that can be regarded as a bridge to cardiac transplantation with limited exceptions. SV patients with Fontan circulation are at risk for various complications including systolic and diastolic ventricular dysfunction which can significantly impact morbidity and mortality [4–7]. Registry data indicate that patients with a single left ventricle (SLV) seem to have a better freedom from late Fontan failure compared to patients with a single right ventricle (in particular hypoplastic left heart syndrome) [3].

Abnormal hemodynamics due to unfavorable volume and/ or pressure load of the SV, as well as the stepwise surgical procedures, might be a cause for SV dysfunction and heart failure [8]. More recently associations between myocardial fibrosis and adverse SV function have been demonstrated [9, 10].

Echocardiography and cardiovascular magnetic resonance (CMR) are the standard imaging modalities to evaluate SV function in patients with a Fontan circulation [11, 12]. Beside traditional techniques, tissue tracking methods such as 2-dimensional (2D) CMR feature tracking (2D-CMR-FT) and 2D speckle tracking echocardiography (2D-STE) have gained popularity to assess global and regional myocardial deformation of the SV [13–15]. However, most studies have assessed a mixed cohort of single left (SLV) and single right ventricle (SRV) patients but only few assessed a uniform population of only SLV patients using 2D-STE [16, 17]. Furthermore, a comparison to healthy controls was only performed in a small cohort in 2 studies [16–18].

For the present study, we hypothesized that ventricular function and myocardial deformation in patients with a SLV are impaired compared to healthy controls. We used 2D-CMR-FT and 2D-STE and analyzed myocardial deformation and ventricular function in a relatively large cohort of SLV patients and healthy controls. In addition, patients with tricuspid atresia (TA) were compared to those with a double inlet left ventricle (DILV) and 2D-CMR-FT results were compared to those from 2D-STE.

## Material and methods

### Patient population

This retrospective study was approved by the ethics committee of the medical faculty of the Christian-Albrechts University Kiel (No. D555/19) and included all 54 SLV patients after Fontan completion, who received a CMR examination as part of a routine clinical follow up during 2010–2019. 35 age-matched healthy controls were included for comparison. For patients who received several CMR examinations during that period, only the most recent dataset was included in our study. The comparison between 2D-CMR-FT and 2D-STE was conducted in datasets in which an echocardiography was performed within 3 months of the CMR examination. To guarantee comparability, echocardiographic studies in patients who underwent cardiovascular surgery or catheter interventions between echocardiography and CMR were excluded.

Age at examination and total cavopulmonary connection (TCPC) as well as gender, weight, height, body surface area (BSA), New York Heart Association (NYHA) functional class, transcutaneous oxygen saturation (SpO_2_) and number of surgical procedures was collected from medical records. Heart rate (HR), cardiac axis and QRS duration was assessed from 12-lead electrocardiograms (ECG).

### Cardiovascular magnetic resonance acquisition and analysis

CMR examinations were performed using a 3 T MRI system (Achieva TX-Series, Philips Healthcare, Best, Netherlands). In 4 older patients a 1.5 T MRI system (Achieva, Philips Healthcare, Best, Netherlands) was used. Patients were sedated using midazolam and propofol according to our clinical protocol, if necessary. Blood Pressure, HR and SpO_2_ levels were monitored during examination. Short-axis, four-chamber and axial cine images were acquired using steady-state free precession or gradient echo pulse sequences. Field of view and slice thickness varied according to patient size (250–400 × 250–400 mm^2^, 5–8 mm).

**Fig. 1:**
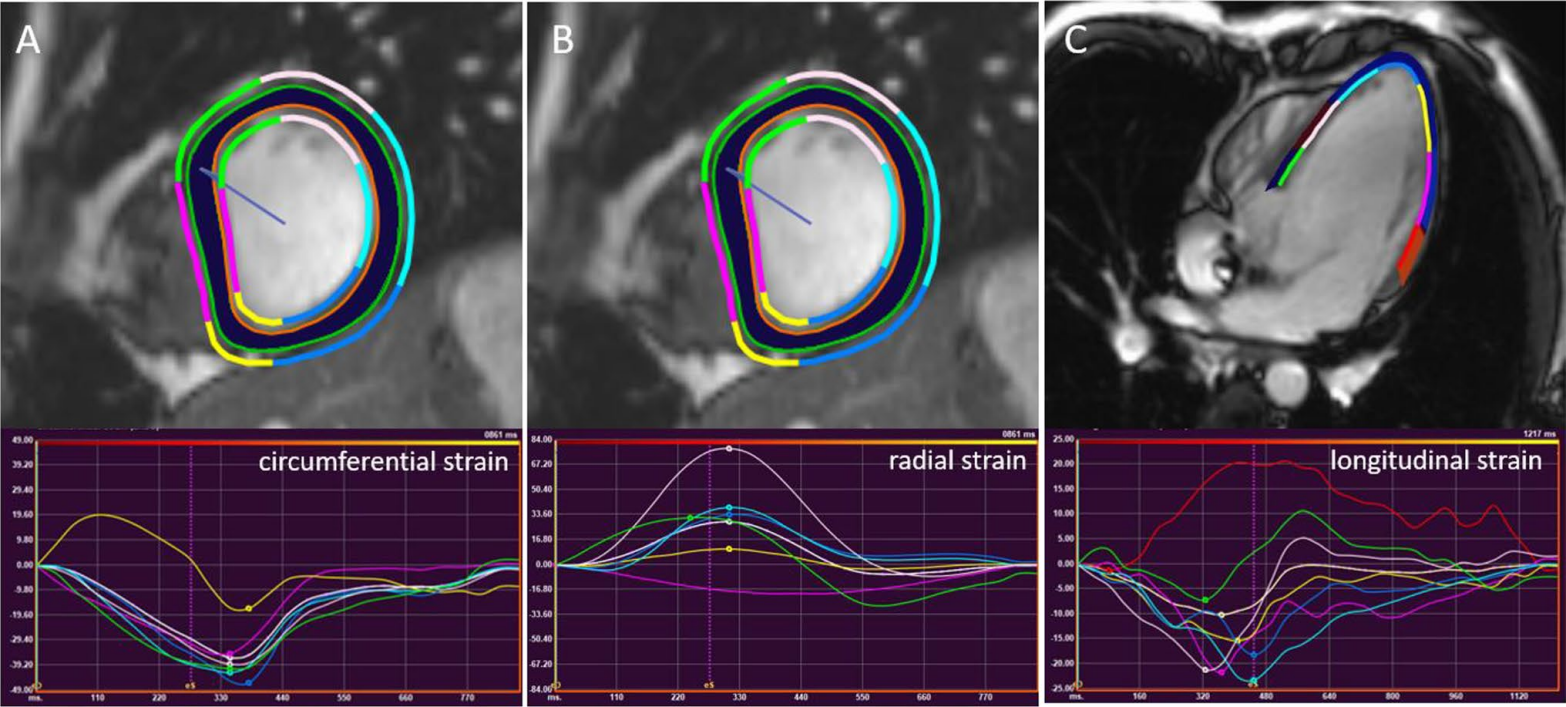
Assessment of LV circumferential (**a**), radial (**b**) and longitudinal (**c**) strain from short axis and long axis images

Volumetric analysis was performed using QMass (Version 8.1, Medis Medical Imaging Systems, BV, Leiden, Netherlands). Left ventricular (LV) end-diastolic and end-systolic volumes (LVEDV, LVESV) were measured from the short-axis images by manual drawing of endocardial and epicardial border at end-diastole and endocardial borders at end-systole (Fig. 1). Papillary muscles and large trabeculations were excluded from the ventricular mass and included into the ventricular volumes. The right ventricular volume was excluded as well. Left ventricular ejection fraction (LVEF), stroke volume and end-diastolic myocardial mass (LVMM) were automatically calculated by the software. Volumes and mass were indexed to BSA.

2D-CMR-FT analysis was undertaken using the dedicated software QStrain Research Edition (Version 2.0, Medis Medical Imaging Systems, BV, Leiden, Netherlands). Global longitudinal strain (GLS) and strain rate (GLSR) were measured in the four-chamber view (Fig. 1). If the examinations lacked a four-chamber-view, we used comparable axial cine images instead (n = 16). Global and regional circumferential strain (CS) and strain rate (CSR) as well as radial strain (RS) and strain rate (RSR) were analyzed from short axis images.

Endocardial and epicardial contours were drawn manually at end-systole, which was defined as the cardiac phase with the smallest LV cavity area. Contours were then tracked automatically by the software during the cardiac cycle. Visual inspection of the epi- and endocardial contours during the cardiac cycle was conducted to evaluate tracking quality and suggested end-diastolic contours were manually adjusted if necessary.

In the four-chamber views, the LV was automatically divided into 7 myocardial segments by the software and peak longitudinal strain and strain rate for each segment was obtained.

In the short axis, the LV was divided into 3 different levels (basal, mid-ventricular and apical) and 16 segments according to the American Heart association 16-segment-model (Fig. 1) [19]. Peak circumferential and radial strain and strain rate values were acquired for each segment.

Global strain (GS) and global strain rate (GSR) values as well as strain and strain rate values for the three ventricular levels were calculated by averaging the peak values of each segment.

### 2-Dimensional speckle tracking echocardiography analysis

Transthoracic echocardiography was performed using a Vivid 7 GE Dimension-System (General Electric Healthcare, Wisconsin, USA). All studies were stored digitally and were therefore available for offline analysis. The data analysis was performed using dedicated STE software (EchoPac, version 113, General Electric Healthcare, Wisconsin, USA) as previously described by our group [15].

### Statistics

Statistical analysis was performed by using a dedicated software (MedCalc statistical software, version 19.5.1, software, Mariakerke, Belgium). Continuous variables were expressed either as mean ± standard deviation if they were normally distributed, or otherwise as median with range. Normal distribution of the data was assessed using the Shapiro Wilk test. Differences between patients and controls as well as between patient subgroups were analysed using the Mann–Whitney-U test. Comparison between extracted mean values using 2D-CMR-FT and 2D-STE was performed using the paired samples t-test. Adjustments for multiple testing were performed and the significant p-value was reduced to 0.003. Bland–Altman plots were constructed to assess the agreement between 2D-CMR-FT and 2D-STE. Associations between variables were evaluated by the Spearman’s rank method and p values of < 0.05 were considered to indicate statistical significance.

## Results

The patient population consisted of 24 patients with TA, 19 patients with DILV and 11 patients with diverse SLV anatomies. Characteristics of all patients and controls are presented in Table 1. Clinical characteristics for patients with TA and DILV are separately shown in Table 2. All patients were examined after TCPC. Median age of the entire patient group was 11.4 years (range 3.1–38.1 years). All except two patients were in NYHA class I.

**Table 1:** Patient characteristics and clinical data

	Patients (n = 56)	Controls (n = 35)	p value*
Age at CMR examination, y	11.4 (3.1–38.1)	12.3 (6.3–25.8)	0.28
Female, n (%)	27 (50)	13 (37)	–
Body height, cm	147.0 (97.5–188.0)	155.0 (121.0–174.0)	0.19
Body weight, kg	38.7 (14.3–93.0)	51.0 (19.0–80.0)	0.05
BSA, m^2^	1.2 (0.6–2.2)	1.4 (0.8–1.9)	0.07
SpO_2_, %	93.0 (78.0–98.0)		
Age at Fontan completion, y	2.7 (1.5–26.3)		
Time since Fontan completion, y	8.7 (1.0–32.2)		
Diagnosis, n (%)			
Tricuspid atresia	24 (44)		
Double inlet left ventricle,	19 (35)		
Atrioventricular septal defect with LV dominance	3 (6)		
Pulmonary atresia	2 (4)		
Other	6 (11)		
Type of Fontan, n (%)			
Intraatrial lateral tunnel	43 (79.6)		
Extracardiac conduit	9 (16.7)		
Fontan-Bjoerk modification	1 (1.9)		
Atriopulmonary connection	1(1.9)		
Fenestration, n (%)			
Open	27 (50)		
Closed/non-fenestrated tunnel	27 (50)		
NYHA functional class, n (%)			
I	52 (96)		
II	2 (4)		

*SpO*_*2*_ oxygen saturation, *y* year

*Comparisons were performed using the Mann–Whitney U test

**Table 2:** Characteristics and clinical data of TA and DILV patients

Parameter	TA (n = 24)	DILV (n = 19)	*p value
Age at CMR examination, y	14.1 (3.1–38.1)	9.4 (3.9–37.0)	0.22
Female, n (%)	12 (50)	9 (47)	–
Body height, cm	158.0 (97.5–188.0)	135.0 (99.0–180.0)	0.17
Body weight, kg	48.9 (14.3–92.0)	39.3 (16.0–93.0)	0. 23
BSA, m^2^	1.5 (0.6–2.2)	1.2 (0.7–2.2)	0.22
Age at Fontan completion, y	2.7 (2–7.7)	2.5 (1.5–26.3)	0.18
Time since Fontan completion, y	9.3 (1.0–32.2)	7.6 (1.3–24.6)	0.34
Type of Fontan, n (%)			
Intraatrial lateral tunnel	20 (83.3)	15 (78.9)	
Extracardiac conduit	3 (12.5)	4 (21.1)	
Fontan-Bjoerk modification	1 (4.2)	–	
Fenestration, n (%)			
Open	8 (33.3)	13 (68.4)	
Closed/non-fenestrated tunnel	16 (66.7)	6 (31.6)	
NYHA functional class, n (%)			
I	23 (95.8)	18 (94.7)	
II	1 (4.2)	1 (5.3)	

### Global and regional myocardial deformation and function

GLS, GLSR and LV volume were determined using a 2D-CMR-FT analysis in all 54 patients. Global and regional CS, CSR, RS and RSR values were acquired in 53 patients in the short axis view. In one patient with DILV the short axis stack did not cover the entire LV.

Global systolic function derived from CMR volumetry was preserved in 52% (n = 28) of SLV patients with an LVEF of ≥ 55%. However, compared to the healthy controls LVEF was reduced (Table 3). Median indexed left ventricular end-systolic volume (LVESVi) from CMR analysis was significantly higher in patients compared to controls whereas indexed left ventricular end-diastolic volume (LVEDVi) and indexed left ventricular myocardial mass (LVMMi) were not significantly different. There was no difference between TA and DILV patients regarding indexed LV volumes, LVEF and LVMMi, all derived by CMR (Table 3). Patients with an age > 20 years had higher values for LVEDVi, LVESVi and LVMMi as well as a reduced LVEF (Table 4).

**Table 3:** Volumetric data from cardiovascular magnetic resonance imaging

Parameter	SLV (n = 53)	Controls (n = 35)	*p value	TA (n = 24)	DILV (n = 18)	*p value
LVEDVi (ml/m^2^)	81.3 [70.8; 88.9]	74.8 [68.1; 84.3]	0.12	77.7 [73.7; 84.7]	87.3 [81.9; 101.5]	0.06
LVESVi (ml/m^2^)	35.9 [28.6; 44.0]	28.3 [25.5; 33.8]	**0.0009**	34.5 [28.7; 43.9]	40.6 [35.9; 45.9]	0.19
LVSVi (ml/m^2^)	43.9 [40.2; 48.9]	45.5 [41.9; 52.1]	0.44	43.7 [38.9; 47.3]	47.0 [40.6; 56.9]	0.14
LVEF (%)	55.6 [51.4; 60.1]	61.2 [58.1; 64.7]	**0.0001**	55.1 [49.0; 61.9]	53.1 [51.0; 58.5]	0.67
LVMMi (g/m^2^)	49.7 [43.4; 58.3]	47.2 [42.8; 55.6]	0.41	48.2 [40.1; 60.1]	51.6 [46.3; 55.5]	0.45

Values are presented as median with and 1st and 3rd quartile

*LVEDVi* indexed left ventricular end-diastolic volume, *LVESVi* indexed left ventricular end-systolic volume, *LVSVI* indexed left ventricular stroke volume, *LVEF* left ventricular ejection fraction, *LVMMi* indexed left ventricular myocardial mass

*Comparisons were performed using the Mann–Whitney U test. Statistically significant p values are indicated in bold

**Table 4:** CMR data across age groups in SLV patients

Parameter	< 10 years (n = 23)	10–20 years (n = 24)	> 20 years (n = 7)
GLS (%)	− 15.2 [− 18.5; − 13.7]	− 16.0 [− 18.7; − 14.8]	14.6 [− 16.7; − 13.4]
GLSR (1/s)	− 1.3 [− 1.6; − 1.0]	− 1.2 [− 1.4; − 0.9]	− 1.2 [− 1.7; − 0.8]
GCS (%)	− 20.3 [− 21.9; − 17.9]	− 21.6 [− 24.3; − 18.8]	17.1 [− 22.0; − 16.2]
GCSR (1/s)	− 1.2 [− 1.4; − 1.2]	− 1.2 [− 1.3; − 1.0]	− 1.1 [− 1.4; − 0.9]
GRS (%)	51.2 [45.8; 60.8]	53.1 [42.4; 61.9]	44.5 [38.9; 62.1]
GRSR (1/s)	2.5 [2.2; 2.9]	1.9 [1.6; 2.3]	1.8 [1.7; 2.1]
LVEDVi (ml/m^2^)	76.5 [66.8; 86.6]	82.5 [73.9; 93.4]	85.0 [74.2; 104.8]
LVESVi (ml/m^2^)	35.9 [27.0; 43.6]	32.1 [28.1; 41.8]	44.5 [35.6; 53.1]
LVSVi (ml/m^2^)	41.2 [38.3; 46.2]	46.7 [42.0; 52.2]	44.8 [35.9; 53.0]
LVEF (%)	55.3 [51.5; 58.5]	58.5 [52.3; 62.4]	51.1 [43.1; 52.1]
LVMMi (g/m^2^)	47.7 [42.1; 55.6]	51.5 [43.4; 62.6]	53.7 [44.3; 71.9]

Values are presented as median with interquartile range

*GLS* global longitudinal strain, *GLSR* global longitudinal strain rate, *GCS* global circumferential strain, *GCSR* global circumferential strain rate, *GRS* global radial strain, *GRSR* global radial strain rate, *LVEDVi* indexed left ventricular end-diastolic volume, *LVESVi* indexed left ventricular end-systolic volume, *LVSVI* indexed left ventricular stroke volume, *LVEF* left ventricular ejection fraction, *LVMMi* indexed left ventricular myocardial mass

**Fig. 2:**
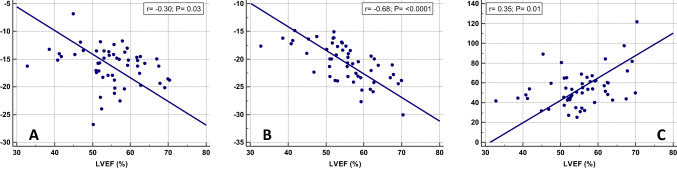
Graphs showing the associations between LVEF and global longitudinal strain (**a**) as well as between circumferential and radial strain (**b** and **c**) in the entire patient cohort

Median GS and GSR values from 2D-CMR-FT are shown in Table 5. Compared to healthy controls, patients had significantly reduced values for GLS and global circumferential strain (GCS) and global circumferential strain rate (GCSR). There was no difference for GLSR and global radial strain rate (GRSR) as well as for global radial strain (GRS) measured by 2D-CMR-FT between patients and controls. 2D-CMR-FT derived GLS, GCS and GRS correlated with LVEF from CMR volumetry in the entire patient group (Fig. 2). When comparing TA and DILV patients there was no statistically significant difference for GLS, GLSR, GCS, GCSR, GRS and GRSR measurements from 2D-CMR-FT between both groups. Patients > 20 years of age had lower values for GLS, GCS, GRS, GCSR and GRSR (Table 4).

**Table 5:** Comparison of 2D-CMR-FT data between patients and controls as well as between patients with TA and DILV

Parameter	Single LV (n = 54)	Controls (n = 35)	*p value	TA (n = 24)	DILV (n = 18)^†^	*p value
GLS (%)	− 15.8 [− 18.3; − 14.2]	− 24.1 [− 26.3; − 22.5]	** < 0.0001**	− 15.3 [− 18.5; − 14.2]	− 16.3 [− 17.6; − 14.5]	0.64
GLSR (1/s)	− 1.2 [− 1.5; − 1.0]	− 1.3 [− 1.4; − 1.1]	0.28	− 1.3 [− 1.5; − 1.0]	− 1.4 [− 1.7; − 1.1]	0.69
GCS (%)	− 20.5 [− 23.3; − 17.7]	− 30.2 [− 33.5; − 29.0]	** < 0.0001**	− 20.0 [− 23.2; − 17.2]	− 19.6 [− 23.2; − 17.2]	0.48
GCSR (1/s)	− 1.2 [− 1.3; − 1.1]	− 1.8 [− 2.0; − 1.6]	** < 0.0001**	− 1.2 [− 1.3; − 1.1]	− 1.2 [− 1.3; − 1.0]	0.78
GRS (%)	51.2 [42.6; 61.8]	55.9 [46.2; 62.4]	0.47	49.7 [44.1; 58.0]	53.6 [43.7; 64.7]	0.46
GRSR (1/s)	2.2 [1.8; 2.7]	2.1 [1.9; 2.6]	0.90	2.2 [1.9; 2.5]	2.3 [1.9; 2.5]	0.67

*GLS* global longitudinal strain, *GLSR* global longitudinal strain rate, *GCS* global circumferential strain, *GCSR* global circumferential strain rate, *GRS* global radial strain, *GRSR* global radial strain rate

*Comparisons were performed using the Mann–Whitney U test. Statistically significant p values are indicated in bold

^†^GLS- and GLSR-Average were measured in 19 patients. Values are presented as median with interquartile range

**Fig. 3:**
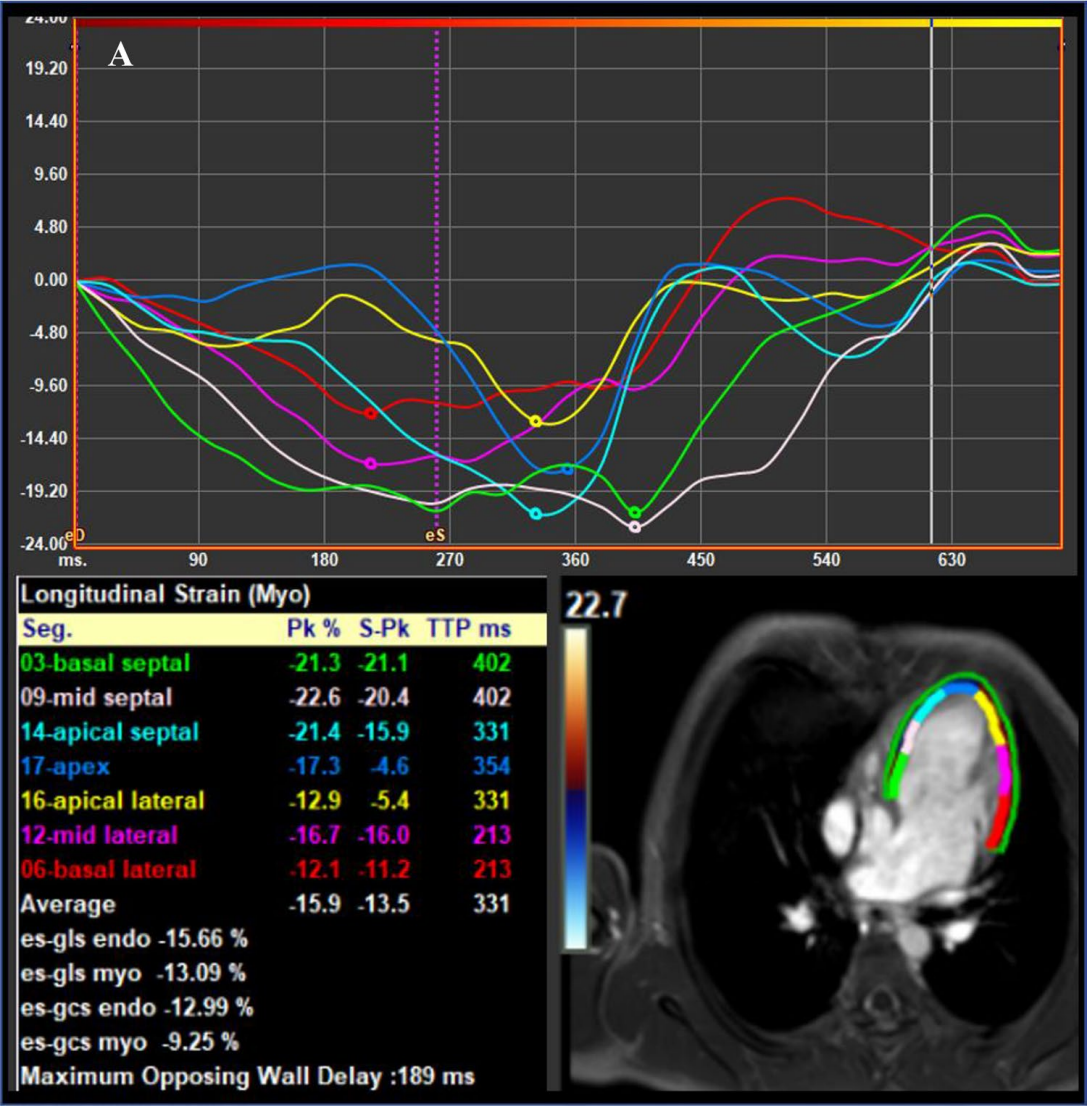

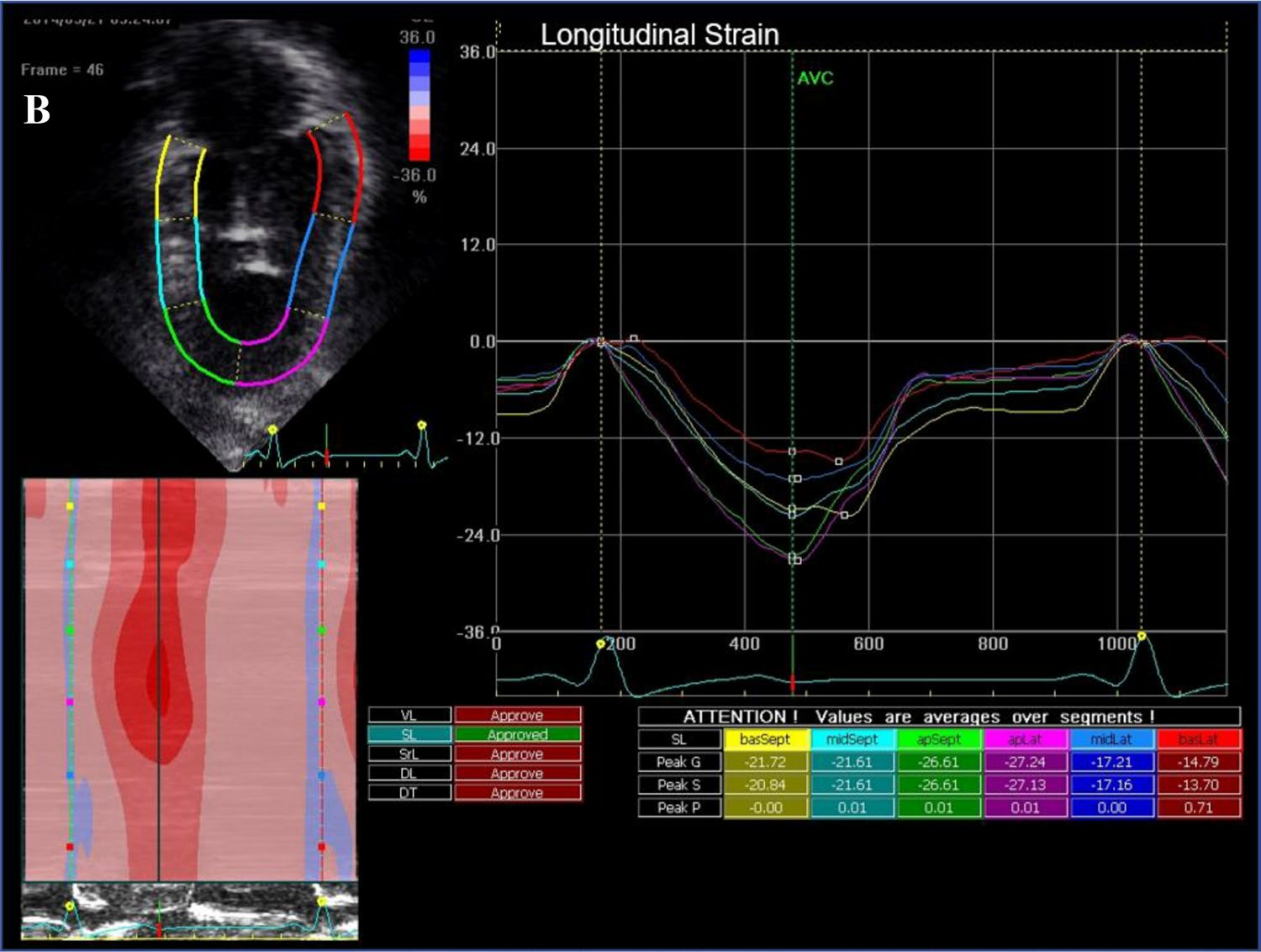
Example of 2D-CMR-FT (**a**) and 2D-STE (**b**) analysis in a patient with tricuspid atresia after TCPC completion

**Fig. 4:**
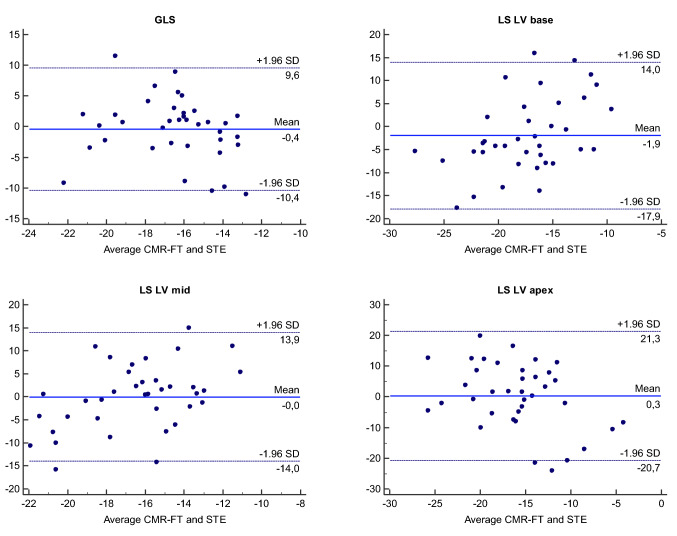
Bland–Altman-Plots comparing 2D-CMR–FT and 2D-STE. Horizontal solid lines represent the mean difference between both analysis techniques and the dashed lines indicate the mean ± 1.96 standard deviation of the difference

Out of the 54 patients, 44 underwent echocardiography within 3 months of the CMR study. 7 patients were excluded from the analysis due to poor image quality or inability to visualize the entire LV. Comparison between 2D-CMR-FT and 2D-STE are illustrated in Table 6 as well as in Fig. 3. Mean GLSR by 2D-CMR-FT was higher than by 2D-STE (− 1.2 ± 0.4 1/s vs. − 0.9 ± 0.2 1/s, p < 0.001). No difference was found for GLS and longitudinal strain at basal, mid and apical level. Bland–Altman-Plots are demonstrated in Fig. 4 and show that the agreement for the GLS measurements using 2D-CMR-FT and 2D-STE was acceptable.

**Table 6:** Comparison of global and regional longitudinal deformation parameters measured by 2D-CMR-FT and 2D-STE

Myocardial deformation	CMR-FT (n = 38)	2D-STE (n = 37)	*p value
GLS (%)	− 16.7 ± 3.2	− 16.3 ± 3.9	0.63
GLSR (1/s)	− 1.3 ± 0.3	− 0.9 ± 0.2	** < 0.0001**
LS LV base (%)	− 18.3 ± 7.0	− 16.4 ± 4.3	0.16
LS LV mid-cavity (%)	− 16.5 ± 5.5	− 16.5 ± 3.4	0.98
LS LV apex (%)	− 15.7 ± 5.7	− 16.0 ± 8.6	0.88

Statistically significant p values are indicated in bold

*GLS* global longitudinal strain, *GLSR* global longitudinal strain rate, *LS LV base* left ventricular longitudinal strain at the basal level, *LS LV mid-cavity* left ventricular longitudinal strain at the mid-ventricular level, *LS LV apex* left ventricular longitudinal strain at the apex

## Discussion

One of strengths of the present study is that a relatively large cohort of SLV patients (n = 54) were included, and that they were compared to healthy controls. Although median LVEF, GLS and GCS from 2D-CMR-FT were reduced compared to controls, most patients had a normal NYHA functional class.

### Myocardial deformation and function in single left ventricle patients compared to controls

Strain analyses using 2D-CMR-FT and 2D-STE in SV patients have been performed by other groups, but most studies included small and mixed patient cohorts (17, 20) [17, 20]. The present study, however, included a relatively large cohort of SLV patients (n = 54) and found significantly reduced values for GLS, GCS and GCSR by 2D-CMR-FT compared to healthy controls. Hu et al. observed significantly reduced GCS and GRS values in Fontan patients compared to controls using CMR [21]. Different to our study, they only included patients with a preserved LVEF (> 55%) and concluded that global and regional circumferential strains could be used for early detection of abnormal myocardial function [21]. That strain values might be impaired before the ejection fraction (EF) is compromised has been demonstrated also in various other patient groups [22, 23] and it has been shown that a preserved EF might be explained mathematically through geometric factors [24].

More than 50% of our SLV patients had a preserved LVEF measured by CMR volumetry but compared to controls LVEF in patients was significantly reduced. Similar findings have been reported by Singh et al. in a small (n = 16) SLV patient cohort [18]. They found a lower LVEF and larger volumetric indices using CMR in pediatric TA patients compared to healthy subjects [18]. Other groups found a reduced LVEF, however they also included patients with a with SRV [14]. A reduction in LVEF in SLV patients compared to controls might be explained by different hemodynamics in some patients and by a heterogeneity in myocardial function in SLV patients [18, 20]. Moreover, an abnormal myoarchitecture as reported in TA patients has to be considered [25].

We were able to show that LVEF from CMR data in SLV patients correlates with GCS, GLS and GRS measured by 2D-CMR-FT. Other groups have shown similar relationships between EF and strain values [24, 26]. Nevertheless, correlations between myocardial deformation parameters and EF are a matter of debate. Lipiec et al. suggested a non-linear hemi-ellipsoid model to explain the association between systolic GLS and LVEF [27]. More recently a mathematical model has been introduced describing the relationship between LVEF, GCS and GLS [28]. In this model a reduction in LVEF would correspond to reduced GCS and GLS values [28].

### Comparison between tricuspid atresia and double inlet left ventricle patients

To our knowledge, no study has compared LV myocardial deformation and function in TA and DILV patients using CMR imaging. Our findings do not suggest any major difference in myocardial deformation, function and size between these two entities. An impaired left ventricular function in patients with TA compared to DILV was found in a cardiac catheterization study by Redington et al. Unfortunately, these results are not comparable with our data from a technical point of view (different imaging modalities) [29].

### Comparison between 2-dimensional cardiovascular magnetic resonance feature tracking and 2-dimensional speckle tracking echocardiography

In our study we found clinically acceptable agreement between 2D-CMR-FT and 2D-STE, however, only 37 echocardiographic examinations could be analyzed. Schmidt et al. analyzed a mixed cohort of adult Fontan patients including both SLV and SRV patients. They highlighted the fact that 2D-CMR-FT allows analyzing all myocardial segments whereas STE is commonly limited by the acoustic windows [14]. Similarly, in our study we had to exclude seven echocardiographic studies because of poor image quality but all CMR examinations were suitable for strain analyses. A study from Ghelani et al. assessed the reproducibility of strain measurements in Fontan patients using 2D-CMR-FT and 2D-STE. Their results suggested that deformation analyses from different modalities should not be mixed [13]. Different to them we did not perform intra-modality reproducibility analyses and we are therefore unable to draw a similar conclusion. However, since 2D-CMR-FT was possible in all SLV patients compared to 2D-STE and that it has become more easily available for routine CMR analyses, we believe that 2D-CMR-FT is a good alternative to 2D-STE. Furthermore, CMR reference values for LV strain values in children and adults exist and can be used for comparison [30, 31].

### Study limitations

The retrospective design of the study implies some limitations. First, in some patients certain CMR data sets were missing and were therefore not available for analysis. In addition, GLS was only measured from the 4-chamber view or axial cine images and this might have impacted our strain results.

Future studies are needed to evaluate the fate of the LV in SLV patients during follow up.

The number of healthy controls was smaller, but since both groups were age-matched this could not influence the study findings.

Finally, we did not perform an intermodality reproducibility analysis for CMR-FT and 2D-STE.

## Conclusions

Most SLV patients had a normal NYHA functional class and 52% of patients had a preserved, CMR derived, LVEF (≥ 55%). However, compared to controls, LVEF, GLS, GCS and GCSR measured by CMR were reduced. Our results suggest that LV deformation and function in SLV patients may behave differently compared to a normal LV in healthy subjects. Follow up studies evaluating the fate of the LV in SLV patients are needed. 2D-CMR-FT might be a suitable modality in this setting.

## Data Availability

All data and materials support the published claims and comply with field standards.
